# Palmitoylethanolamide Dampens Reactive Astrogliosis and Improves Neuronal Trophic Support in a Triple Transgenic Model of Alzheimer's Disease: *In Vitro* and *In Vivo* Evidence

**DOI:** 10.1155/2018/4720532

**Published:** 2018-01-16

**Authors:** Maria Rosanna Bronzuoli, Roberta Facchinetti, Luca Steardo, Adele Romano, Claudia Stecca, Sergio Passarella, Luca Steardo, Tommaso Cassano, Caterina Scuderi

**Affiliations:** ^1^Department of Physiology and Pharmacology “V. Erspamer”, Sapienza University of Rome, Rome, Italy; ^2^Department of Psychiatry, University of Naples SUN, Naples, Italy; ^3^Department of Clinical and Experimental Medicine, University of Foggia, Foggia, Italy

## Abstract

Alzheimer's disease (AD) is a neurodegenerative disorder responsible for the majority of dementia cases in elderly people. It is widely accepted that the main hallmarks of AD are not only senile plaques and neurofibrillary tangles but also reactive astrogliosis, which often precedes detrimental deposits and neuronal atrophy. Such phenomenon facilitates the regeneration of neural networks; however, under some circumstances, like in AD, reactive astrogliosis is detrimental, depriving neurons of the homeostatic support, thus contributing to neuronal loss. We investigated the presence of reactive astrogliosis in 3×Tg-AD mice and the effects of palmitoylethanolamide (PEA), a well-documented anti-inflammatory molecule, by *in vitro* and *in vivo* studies. *In vitro* results revealed a basal reactive state in primary cortical 3×Tg-AD-derived astrocytes and the ability of PEA to counteract such phenomenon and improve viability of 3×Tg-AD-derived neurons. *In vivo* observations, performed using ultramicronized- (um-) PEA, a formulation endowed with best bioavailability, confirmed the efficacy of this compound. Moreover, the schedule of treatment, mimicking the clinic use (chronic daily administration), revealed its beneficial pharmacological properties in dampening reactive astrogliosis and promoting the glial neurosupportive function. Collectively, our results encourage further investigation on PEA effects, suggesting it as an alternative or adjunct treatment approach for innovative AD therapy.

## 1. Introduction

Alzheimer's disease (AD) accounts for more than 80% of dementia cases worldwide in elderly people and leads to the progressive loss of mental, behavioral, and learning abilities and to functional decline [1]. Histopathologically, AD is characterized by two major protein deposits affecting mainly hippocampal and cortical regions: extracellular neuritic *β*-amyloid peptide (A*β*), which induces the creation of senile plaques (SPs), and the production of intracellular neurofibrillary tangles (NFTs) due to tau hyperphosphorylation that occupies much of the cytoplasm of pyramidal neurons [2, 3]. The presence of abnormally activated microglia and astrocytes is a feature of AD of more recent discovery [4]. It is followed by an intense inflammation, closely associated with amyloid deposits in the brain parenchyma [4, 5]. Indeed, studies of post mortem brain tissues from AD patients demonstrated the presence of a generalized astrogliosis, mainly manifested by astrocytic dysfunction, detectable by an increased expression of both glial fibrillary acidic protein (GFAP) and S100B, and accompanied by an increased production of proinflammatory mediators [6]. Many authors name this complex phenomenon as reactive astrogliosis [6, 7]. GFAP is a specific cytoskeletal marker, whose expression is higher during astrogliosis [8]. S100B is a neurotrophin that, at physiological concentrations (nanomolar), exerts prosurvival effects on neurons and stimulates neurite outgrowth [9]. However, at higher (micromolar) concentrations, S100B becomes neurotoxic, promoting inflammation and neuronal apoptosis [10]. A*β* itself induces the expression of proinflammatory cytokines by glial cells [11] and the induction of proinflammatory enzymes, such as the inducible nitric oxide synthase (iNOS) and the isoenzyme cyclooxygenase type-2 (COX-2). Several lines of evidence suggest that all these factors may contribute to neuronal dysfunction and cell death, either alone or in concert [12]. The reactive astrogliosis has the initial intent of defence of removing injurious stimuli. However, if this phenomenon goes beyond physiological control, it may cause several detrimental effects. Under these circumstances, both neuronal and synaptic loss are detectable, because structural and functional modifications of neurons and astrocytes occur [13, 14]. Alterations of the neuronal marker microtubule-associated protein 2 (MAP-2), as well as modifications of the neurotrophin brain-derived neurotrophic factor (BDNF) content, have also been demonstrated [15, 16]. Considering the crucial actions of BDNF, especially in controlling neuronal survival, differentiation, neurotransmitter release, dendritic remodeling, axon growth, and synaptic plasticity [17, 18], the detrimental consequences of its alterations by reactive astrogliosis may be dramatic.

Based on this evidence, it is reasonable to assume that an early combination of neuroprotective and anti-inflammatory treatments may represent an efficacious approach to counteract AD. In this context, palmitoylethanolamide (PEA), an endogenous lipid mediator, seems to be a promising pharmacological agent. The anti-inflammatory and neuroprotective effects of PEA, as well as its ability to attenuate memory impairment in surgical models of AD, have already been demonstrated [15, 19–22].

In this work, we provide novel evidence on the ability of PEA to counteract reactive astrogliosis and neuronal impairment both *in vitro* and *in vivo*. For the *in vitro* studies, we used primary cortical neurons and astrocytes from 3×Tg-AD mice, a triple transgenic model of AD currently considered the closest to the familial human disease, and from wild-type littermates (non-Tg). The same AD model was used for the *in vivo* experiments, in which male 3-month-old 3×Tg-AD and sex- and age-matched non-Tg mice were subcutaneously implanted with a pellet, releasing either ultramicronized-PEA (um-PEA) or placebo, for three months. This treatment schedule was designed to reproduce a chronic treatment (as needed for this type of disease), administered starting from the early stage of the AD pathology.

*In vitro* results highlighted an intense activation and inflammation in primary 3×Tg-AD astrocytes, as well as the ability of PEA to counteract them and promote neuronal viability. Moreover, *in vivo* biochemical experiments demonstrated that chronic um-PEA treatment resulted in a beneficial control of the astrocyte activation and neuroinflammation. In addition, um-PEA interestingly increased BDNF levels, confirming its neuroprotective/neurotrophic effects.

Our results confirm the therapeutic potential of PEA, demonstrating its ability to counteract some of the detrimental effects occurring in AD, since the earliest stage of the pathology. PEA is already on the market for the treatment of pain. Therefore, these observations, in addition to the information regarding its safety and tolerability also in humans, prompt us to hypothesize a rapid translation into clinical practice.

## 2. Materials and Methods

All the procedures involving animals were conducted in conformity with the guidelines of the Italian Ministry of Health (D.L. 26/2014) and performed in compliance with the European Parliament directive 2010/63/EU.

### 2.1. Animals and Experimental Design

3×Tg-AD mice [23] expressing APP_swe_, PS1_M146V_, and tau_P301L_ human transgenes were compared to non-Tg littermates. The background strain of 3×Tg-AD mice was C57BL6/129SvJ hybrid [23]. Animals were group housed and raised in controlled conditions (22 ± 2°C temperature, 12 h light/12 h dark cycle, 50%–60% humidity) in an enriched environment, with food and water ad libitum.

For *in vitro* experiments, we used newborn mice at postnatal day (PND) 1 or 2. Astrocytes were isolated from both non-Tg (total pups used = 12) and 3×Tg-AD (total pups used = 24) mice. Neurons were isolated from 3×Tg-AD mice (total pups used = 12).

For *in vivo* experiments, 3-month-old male non-Tg (*n* = 18) and 3×Tg-AD (*n* = 18) mice were used. Animals were surgically implanted with a 90-day-release pellet containing either 28 mg um-PEA, a formulation that improves its bioavailability [24], or placebo (catalogue number NX-999 and NC-111, resp.; Epitech Group SpA). Both pellets were made by Innovative Research of America (Sarasota, Florida) that homogeneously distributed um-PEA in the matrix, keeping its original crystalline form of micrometric size. Therefore, mice received 10 mg/kg/day for 3 consecutive months. Experimental dosage was chosen according to literature [25, 26].

To subcutaneously implant the pellet, mice were anesthetized with ketamine hydrochloride (1 mg/10 g) and xylazine (0.1 mg/10 g). After shaving the shoulder blades, the implantation area was sterilized with 70% alcohol. A dorsal midline incision of 1-2 cm was executed to create a subcutaneous pocket with a blunt probe. One pellet, containing um-PEA or placebo, was placed into the pocket and the surgical incision was closed with sterile absorbable sutures. Non-Tg and 3×Tg-AD mice were then left in their home cages for the next three months, and their weight monitored daily. No weight differences among all experimental groups were detected (data not shown). At the end of the chronic treatment, 6-month-old mice were killed by decapitation, and their brains were rapidly excised and either immediately frozen on dry ice for the immunofluorescence experiments or freshly dissected to isolate the frontal cortex (FC) for RT-PCR and Western blot analyses.

**Figure 1:**
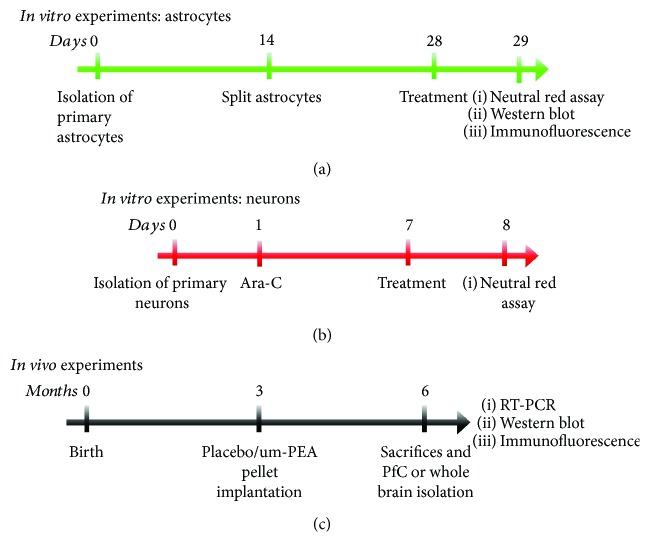
Study designs. Schematic representation of the experimental designs in (a) primary 3×Tg-AD and non-Tg astrocytes, (b) primary 3×Tg-AD neurons, and (c) *in vivo* experiments in 3×Tg-AD and non-Tg mice.

The experimental timelines are summarized in Figure 1.

### 2.2. Astroglial Primary Cultures

Astroglial primary cultures were obtained as previously described [27]. Cortices were isolated from non-Tg and 3×Tg-AD newborn mice (PND 1 or 2) sacrificed by decapitation. Tissues were manually homogenized in Dulbecco's modified Eagle's medium (DMEM) supplemented with 5% inactivated fetal bovine serum (FBS), 100 U/ml penicillin and 100 *μ*g/ml streptomycin and then chemically dissociated with a solution containing 0.25% trypsin, 0.2% ethylenediaminetetraacetic acid (EDTA), and 0.2 mg/ml of DNAse I (all from Sigma-Aldrich, Milan, Italy) to obtain single cells. After a centrifugation at 403 ×g for 5 minutes, the medium was replaced, and surviving cells were counted using a Burker chamber with a 0.2% trypan blue solution and seeded at a density of 3 × 10^−6^ cells/75 cm^2^ flask. Cell cultures were at +37°C in humidified atmosphere containing 5% CO_2_. DMEM supplemented with 20% inactivated FBS and 100 U/ml penicillin, and 100 *μ*g/ml streptomycin was replaced 24 h and one week after isolation. Approximately 14-15 days after the dissection, when a monolayer of cells was created, astrocytes were separated from microglia by mechanical shacking. Then, astrocytes were detached from the plates with a solution containing 0.25% trypsin and 0.2% EDTA and then seeded into 10 cm diameter petri dishes at a density of 1 × 10^6^ cells/dish for Western blot analysis, into eight chambers polystyrene culture slides at a density of 3 × 10^4^ cells/chamber (Thermo Fisher Scientific, MA, USA) for immunofluorescence, or into 24-well plates at a density of 1 × 10^5^ cells/well for neutral red viability assay. Experiments were performed 28 days after cells isolation, when astrocytes are considered completely mature [22].

### 2.3. Neuronal Primary Cultures

Cortices from newborn 3×Tg-AD mice (PND 1 or 2) were used to obtain primary neuronal cultures as previously described [20]. Multiwells were previously coated with poly-L-lysine (Sigma-Aldrich) to allow neurons to adhere to the bottom of the wells. Mice were sacrificed by decapitation and cerebral cortices dissected in Hank's balanced salt solution (HBSS) containing 25 mM 4-(2-hydroxyethyl)-1-piperazineethanesulfonic acid (Hepes), 100 U/ml penicillin, and 100 *μ*g/ml of streptomycin in cold conditions. Tissues were mechanically and chemically homogenized in a solution containing 0.25% trypsin, 0.2% EDTA, and 0.2 mg/ml of DNAse I (all from Sigma). After a centrifugation at 403 ×g for 5 minutes, cells were suspended in neurobasal media supplemented with 2% B27, 100 U/ml penicillin, and 100 *μ*g/ml streptomycin. Then, they were counted using a Burker chamber while suspended in 0.2% trypan blue solution and then seeded in 24-well multiplates at a density of 5 × 10^−5^ cells/well. Neuronal cultures were maintained at +37°C in humidified atmosphere containing 5% CO_2_. Twenty-four hours later, cells were treated with 10 *μ*M of cytosine arabinoside (ara-C) to suppress glial cell growth. One week later, primary 3×Tg-AD-derived neurons were treated with PEA.

### 2.4. Chemicals and Cell Treatments

Mature astrocytes and neurons derived from 3×Tg-AD mice were treated with PEA (Epitech Group SpA, Saccolongo, Italy), at three different concentrations (0.01, 0.1, and 1 *μ*M), chosen according to our previous works [19, 20, 22]. PEA was suspended in 5% pluronic F-68 (Sigma-Aldrich) and solubilized in 95% DMEM. First, we added pluronic F-68 to PEA powder and sonicated the emulsion for 20 min protecting it from light, and then we included DMEM to complete the solution. Viability assay, Western blot analysis, and immunofluorescence were performed 24 h after treatments.

### 2.5. Analysis of Astrocyte and Neuronal Viability by Neutral Red Uptake Assay

Astrocyte and neuronal viability was tested 24 h after treatment by neutral red uptake assay, as previously described [27]. Cells were incubated with a neutral red working solution, containing 50 *μ*g/ml in Ca^2+^ and Mg^2^-free PBS (Sigma-Aldrich), for 3 h at +37°C. Cells were then rinsed in Ca^2+^- and Mg^2^-free PBS and the dye removed from the inside of the cells through a rinse in destaining solution (ethanol : deionized water : glacial acetic acid, 50 : 49 : 1 *v*/*v*). The absorbance, whose value is proportional to the number of living cells, was read at 540 nm using a microplate spectrophotometer (Epoch, BioTek, Winooski, VT, USA). The values obtained were referred to control medium-exposed cultures (CTRL) and expressed as percentage variation of CTRL. Three independent experiments were performed in triplicate.

### 2.6. Immunofluorescence

Both primary astrocytes, 24 h after treatment, and non-Tg and 3×Tg-AD coronal slices (12 *μ*m thickness), deriving from 6-month-old mice containing the FC, were rinsed in PBS and postfixed for 10 minutes at +4°C with 4% paraformaldehyde (PFA) prepared in PBS. Then, samples were blocked with 1% bovine serum albumin (BSA) prepared in PBS/0.25% triton X-100 for 90 minutes at room temperature. Cells were incubated overnight at +4°C in 0.5% BSA in Tris-buffered saline (TBS)/0.25% triton X-100 solution containing the primary antibodies rabbit anti-GFAP (1 : 1000, Abcam, Cambridge, USA) or rabbit anti-S100B (1 : 250, Novus Biologicals, Littleton, CO, USA). FC slices, instead, were incubated overnight at +4°C with mouse anti-MAP2 (1 : 250, Novus Biologicals, Littleton, CO, USA) in 0.5% BSA in TBS/0.25% triton X-100 solution. The following day, cells and tissues were thoroughly rinsed in PBS and then incubated for 2 hours at room temperature with the appropriate secondary antibody (fluorescein- (FITC-) conjugated AffiniPure goat anti-rabbit IgG (H+L), rhodamine- (TRITC-) conjugated AffiniPure goat anti-rabbit IgG (H+L), 1 : 200, or rhodamine- (TRITC-) conjugated AffiniPure goat anti-mouse IgG (H+L); Jackson ImmunoResearch, Suffolk, UK). Nuclei were stained with 4′,6-diamidino-2-phenylindole, dihydrochloride (DAPI) (1 : 75000, Sigma-Aldrich) in 0.5% BSA in TBS/0.25% triton X-100, added to the solution of the secondary antibodies. Samples were rinsed with PBS and coverslipped using Fluoromount aqueous mounting medium (Sigma-Aldrich). Experimental conditions are summarized in Table 1.

Pictures were captured with a wide-field microscope (Eclipse E600; Nikon Instruments, Rome, Italy) and densitometric analysis performed using ImageJ software. Data are expressed as ratio (Δ*F*/*F*_0_) of the difference between the mean of fluorescence sample and its background (Δ*F*) and the nonimmunoreactive regions (*F*_0_). To prevent the observation of differences among experimental groups due to artifacts, the exposure parameters, such as gain and time, were kept constant during image acquisitions. For each analysis, three replicates were used, and at least three independent experiments were performed.

### 2.7. RNA Isolation and RT-PCR

Total mRNA from FC of both non-Tg and 3×Tg-AD mice was extracted using the NZY total RNA isolation kit (NZYTech, Lisboa, Portugal) following the manufacturer's protocol. Total mRNA was quantified by Nanodrop 1000 spectrophotometer (Thermo Fisher Scientific, MA, USA). Revers transcription of 1 *μ*g mRNA was performed to obtain cDNA adding oligo(dT) and random primers to the first-strand cDNA synthesis kit (NZYTech, Lisboa, Portugal). All PCRs were performed using the supreme NZYTaq DNA polymerase (NZYTech, Lisboa, Portugal) in the presence of specific primers (Sigma-Aldrich) for the target genes: GFAP, GAPDH, S100B, iNOS, and COX-2. GAPDH was used as reference gene. Three independent experiments were performed in triplicate. Primer sequences and PCR details are reported in Table 2.

### 2.8. Protein Extraction and Western Blot Analysis

Western blot analysis was performed on protein extracts obtained from primary astrocytic cultures as well as from FC samples, as previously described [15]. Samples were suspended in ice-cold hypotonic lysis buffer containing 50 mM Tris/HCl pH 7.5, 150 mM NaCl, 1 mM EDTA, 1% triton X-100, 1 mM phenylmethylsulfonyl fluoride (PMSF), 10 *μ*g/ml aprotinin, and 0.1 mM leupeptin (all from Sigma-Aldrich). After 40 min of incubation at +4°C, homogenates were centrifuged at 18440 ×g for 30 min and the supernatant collected and stored in aliquots at −80°C until use. An equivalent amount of each sample (50 *μ*g), calculated by Bradford assay, was resolved through 12% acrylamide SDS-PAGE precast gels (Bio-Rad Laboratories, Segrate, Italy). Then, with a trans-blot SD semidry transfer cell (Bio-Rad Laboratories), proteins were transferred onto nitrocellulose membranes that were then blocked with 5% no-fat dry milk powder or 5% BSA in TBS 0.1% Tween 20 (TBS-T) (Tecnochimica Moderna, Rome, Italy) for 1 h before overnight incubation at +4°C with the appropriate primary antibodies. After appropriate rinses in 0.05% TBS-T, membranes were incubated for 1 h at room temperature with a specific secondary horseradish peroxidase- (HRP-) conjugated antibody. The experimental conditions are summarized in Table 3.

Immunocomplexes were detected by an ECL kit (GE Healthcare Life Sciences, Milan, Italy), exposed to X-ray film (GE Healthcare Life Sciences, Milan, Italy) and quantified using ImageJ software. Protein expression level of *β*-actin was used as loading control. For each antibody, three replicates were used, and at least three independent experiments were performed.

### 2.9. Statistical Analysis

Analysis was performed using GraphPad Prism software (GraphPad Software, San Diego, CA, USA). Student's *t*-test was used to compare two groups. One-way analysis of variance (ANOVA) was used to determine statistical differences among experimental groups in *in vitro* experiments. *In vivo* results were analyzed by two-way ANOVA, with genotype and treatment as factors. Bonferroni's post hoc test was used upon detection of a main significant effect. Differences between mean values were considered statistically significant when *P* < 0.05.

## 3. Results

### 3.1. 3×Tg-AD Primary Astrocytes Present Reactive Astrogliosis

Reactive astrogliosis is a phenomenon commonly detectable in AD brains and characterized by both astrocyte activation and neuroinflammation [6]. Here, we decided to test parameters connected with such events in both non-Tg and 3×Tg-AD primary astrocytes.

**Figure 2:**
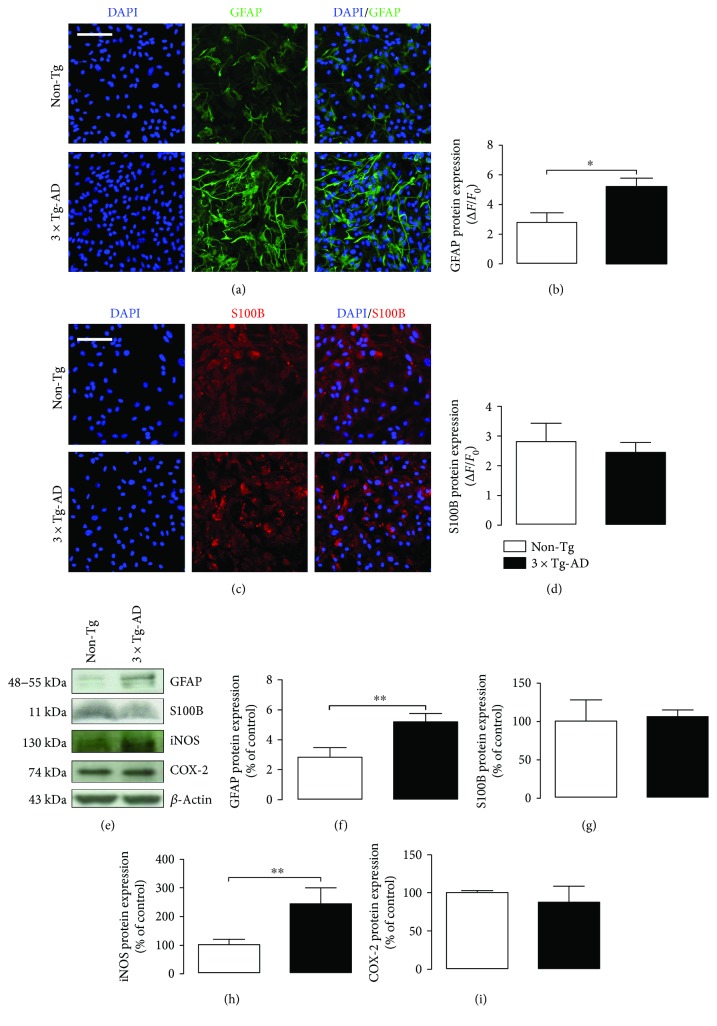
Study of parameters related to reactive astrogliosis in 3×Tg-AD and non-Tg primary astrocytes. (a) Representative fluorescent photomicrographs of GFAP (green) and (b) signal quantification in both non-Tg (white bar) and 3×Tg-AD (black bar) primary astrocytes. (c) Representative fluorescent photomicrographs of S100B (red) and (d) signal quantification in both non-Tg (white bar) and 3×Tg-AD (black bar) primary astrocytes. Nuclei were stained with DAPI (blue). Scale bar is 50 *μ*m. Fluorescence analysis is expressed as Δ*F*/*F*_0_. (e) Representative bands and Western blot densitometric analysis of (f) GFAP, (g) S100B, (h) iNOS, and (i) COX-2. *β*-Actin was used as loading control. Results are expressed as percentage of the mean control value (non-Tg cells). Experiments were performed three times in triplicate. Data are presented as mean ± SEM. The statistical analysis was performed by Student's *t*-test (^∗^*P* < 0.05 and ^∗∗^*P* < 0.01 versus non-Tg group).

To study astrocyte activation, we tested the expression of GFAP, a specific cytoskeletal marker, and S100B, a neurotrophin that when present at high concentrations becomes neurotoxic [6, 28, 29]. Results obtained from both immunofluorescence and Western blot analysis showed a significantly higher GFAP immunoreactivity in primary 3×Tg-AD astrocytes than non-Tg cells (immunofluorescence: *P* < 0.05; Western blot *P* < 0.01) (Figures 2(a), 2(b), 2(e), and 2(f)), while we did not detect changes in S100B signal (*P* > 0.05) (Figures 2(c), 2(d), 2(e), and 2(g)).

A neuroinflammatory environment is mainly characterized by the production and activation of two inducible enzymes: the prostanoid-generating enzyme COX-2 and iNOS [15]. Here, we tested the expression of these two enzymes in non-Tg and 3×Tg-AD primary astrocytes. Results showed that iNOS expression is significantly higher in transgenic-derived primary astrocytes than non-Tg cells (*P* < 0.01) (Figures 2(e) and 2(h)). We did not find any statistical difference in COX-2 expression between the two experimental groups (*P* > 0.05) (Figures 2(e) and 2(i)).

Collectively, these results show that reactive astrogliosis is detectable in mature 3×Tg-AD cortical astrocytes.

### 3.2. PEA Improves Neuronal Viability and Counteracts Reactive Astrogliosis In Vitro

**Figure 3:**
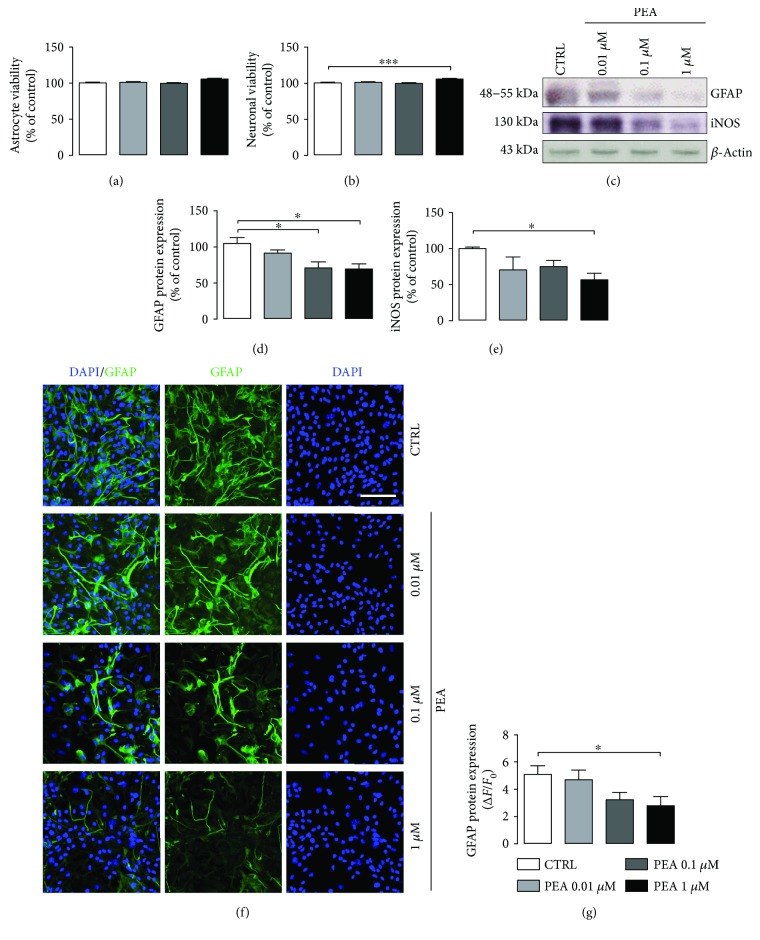
Effect of PEA treatment on astrocyte and neuronal viability and reactive astrogliosis in 3×Tg-AD cells. Evaluation of (a) astrocyte and (b) neuronal viability tested by neutral red uptake assay after 24 h PEA treatment (0.01–0.1–1 *μ*M). (c) Representative immunoreactive signals and Western blot densitometric analysis of (d) GFAP and (e) iNOS. *β*-Actin was used as loading control. Results are expressed as percentage of the mean control value (CTRL). (f) Representative fluorescent photomicrographs of GFAP (green) staining in 3×Tg-AD primary astrocytes. Nuclei were stained with DAPI (blue). Scale bar is 50 *μ*m. (g) Fluorescence analysis is expressed as Δ*F*/*F*_0_. Experiments were performed three times in triplicate. Data are presented as mean ± SEM. The statistical analysis was performed by one-way ANOVA followed by Bonferroni's post hoc multiple comparison test (^∗^*P* < 0.05 and ^∗∗∗^*P* < 0.001 versus CTRL group).

To test whether PEA treatment could have any toxic effect on astrocyte and neuronal viability, we performed the neutral red assay. Results showed that PEA did not affect astrocytes or neuronal viability at all concentrations tested (*P* > 0.05) (Figures 3(a) and 3(b)). Surprisingly, the highest PEA concentration significantly improved neuronal viability (+5.35%; *P* < 0.001) (Figure 3(b)).

Next, we tested the ability of PEA, at different concentrations (0.01, 0.1, and 1 *μ*M), to counteract reactive astrogliosis. Western blot analyses showed that PEA treatment prevented GFAP increase in primary astrocytes derived from newborn 3×Tg-AD mice in a concentration-dependent manner (*P* < 0.05) (Figures 3(c) and 3(d)). Results by immunofluorescence confirmed this trend, although only the highest dose of PEA reached statistical significance (*P* < 0.05) (Figures 3(f) and 3(g)). Moreover, by Western blot, we found that 1 *μ*M PEA was able to significantly reduce iNOS expression (*P* < 0.05) (Figures 3(c) and 3(e)).

These results show that PEA has no toxicity in both astrocytes and neurons from 3×Tg-AD mice, at the concentrations tested; rather, it promotes neuron viability. Moreover, PEA counteracts reactive astrogliosis in mature 3×Tg-AD primary astrocytes.

### 3.3. Chronic Um-PEA Normalizes Reactive Astrogliosis in the Frontal Cortex of 3×Tg-AD Mice

Given the interesting *in vitro* results, and with the aim of further exploring PEA effectiveness in the triple transgenic model of AD, we decided to translate the study *in vivo*. Specifically, we tested the effect of chronic um-PEA treatment on reactive astrogliosis and neuronal functionality in FCs of 6-month-old 3×Tg-AD mice, compared to their age-matched non-Tg littermates.

**Figure 4:**
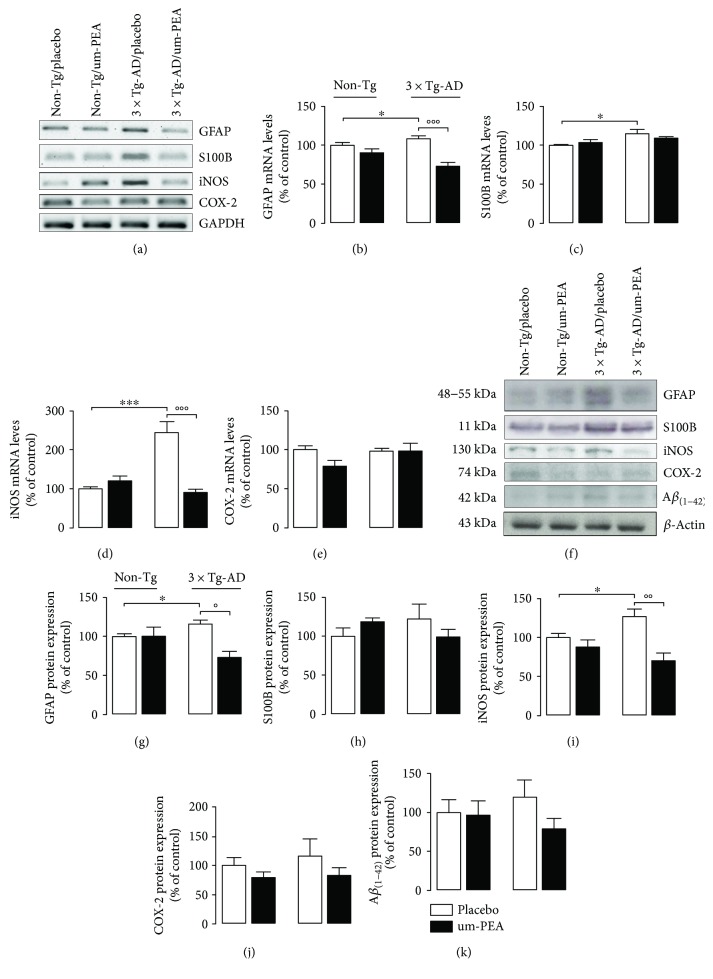
Effect of chronic um-PEA on reactive astrogliosis and A*β*_(1–42)_ expression in the FC of 3×Tg-AD and non-Tg mice. (a) Representative bands from RT-PCR performed in FC homogenates for GFAP, S100B, iNOS, and COX-2, and (b–e) densitometric analysis of the corresponding signals normalized to GAPDH. (f) Representative immunoreactive species and Western blot densitometric analysis of (g) GFAP, (h) S100B, (i) COX-2, (j) iNOS, and (k) A*β*_(1–42)_. *β*-Actin was used as loading control. Results are expressed as percentage of the mean control value (non-Tg/placebo). Experiments were performed three times in triplicate. Data are presented as mean ± SEM. The statistical analysis was performed by two-way ANOVA followed by Bonferroni's post hoc multiple comparison test (^∗^*P* < 0.05 and ^∗∗∗^*P* < 0.001 versus non-Tg/placebo group; °*P* < 0.05, °°*P* < 0.01, and °°°*P* < 0.001 versus 3×Tg-AD/placebo group).

Confirming our *in vitro* observations, we found astrocyte activation in FC of 3×Tg-AD mice when compared with their age-matched non-Tg littermates. Indeed, we found, by RT-PCR and Western blot, an increase of both GFAP and S100B expression in placebo-treated 3×Tg-AD mice in comparison with placebo-treated non-Tg mice (*P* < 0.05) (Figures 4(a), 4(b), 4(c), 4(f), 4(g), and 4(h)). Um-PEA chronic treatment greatly controlled such astrocytic activation, significantly decreasing GFAP mRNA and protein expression (*P* < 0.05) (Figures 4(a), 4(b), 4(f), and 4(g)). Moreover, the two-way ANOVA showed a significant genotype-by-treatment interaction effect on GFAP transcription (*F*_genotype×treatment(1,23)_ = 7.872, *P* = 0.0062) (Figure 4(g)) and expression (*F*_genotype×treatment(1,23)_ = 4.829, *P* = 0.0337) (Figure 4(g)). Surprisingly, um-PEA also induced a trend toward a decrease (−20%) of S100B protein expression in 3×Tg-AD mice compared to placebo-treated 3×Tg-AD ones (Figures 4(a), 4(c), 4(f), and 4(h)).

Regarding the parameters related to the inflammatory process, RT-PCR and Western blot results showed a significant increase of iNOS transcription (*P* < 0.001) (Figures 4(a) and 4(d)) and expression (*P* < 0.05) (Figures 4(f) and 4(i)) in the FC of placebo-treated 3×Tg-AD mice in comparison with placebo-treated non-Tg animals. Interestingly, chronic um-PEA treatment greatly controlled the induced production of this proinflammatory enzyme (RT-PCR: *P* < 0.001; Western blot: *P* < 0.05) (Figures 4(a), 4(d), 4(f), and 4(i)). In addition, two-way ANOVA showed a significant genotype-by-treatment interaction effect in iNOS transcription (*F*_genotype×treatment(1,23)_ = 28.37, *P* < 0.0001) (Figure 4(d)) and protein expression (*F*_genotype×treatment(1,23)_ = 4.894, *P* = 0.0306) (Figure 4(i)). As in *in vitro* results, neither genotype nor treatment induced changes in COX-2 transcript and protein expression (Figures 4(a), 4(e), 4(f), and 4(j)).

Interestingly, using Western blot experiments, we found a trend toward an increase of A*β*_(1–42)_ (+20%) in the FCs of transgenic mice in comparison with the non-Tg animals. This trend, which follows the observed changes in parameters related to astrocyte activation and neuroinflammation, is in line with the evidence available in literature indicating that 6-month-old 3×Tg-AD mice show A*β* overexpression predominantly in FC layers 4 to 5 [23]. Moreover, chronic um-PEA treatment dampened the expression of A*β* in 3×Tg-AD mice, although this failed to reach significance (−34.23%) (Figures 4(f) and 4(k)). Despite this evidence, further experiments will be required to demonstrate the existence of a causative correlation between the reduction of A*β* load and the control of neuroinflammation.

Combined, our results demonstrate the presence of activated astrocytes and a proinflammatory environment in the FCs of 3×Tg-AD mice at 6 months of age. Additionally, we show that chronic um-PEA treatment efficaciously controls these alterations in the AD brain.

### 3.4. Chronic um-PEA Normalizes Astrocyte Support to Neuronal Functionality in the Frontal Cortex of 3×Tg-AD Mice

**Figure 5:**
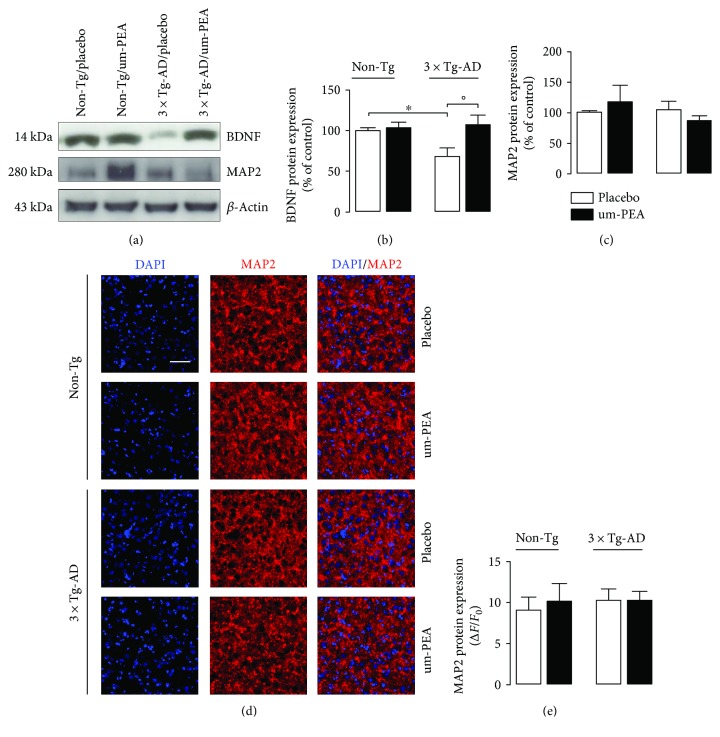
Effect of chronic um-PEA on neuronal support and survival in FC of 3×Tg-AD and non-Tg mice. (a) Representative immunoreactive species and Western blot densitometric analysis of (b) BDNF and (c) MAP2. *β*-Actin was used as loading control. Results are expressed as percentage of the mean control value (non-Tg/placebo). (d) Representative fluorescent photomicrographs of MAP2 (red) staining in FC of 6-month-old non-Tg and 3×Tg-AD mice, placebo- or um-PEA-treated. Nuclei were stained with DAPI (blue). Scale bar is 50 *μ*m. (e) Fluorescence analysis is expressed as Δ*F*/*F*_0_. Experiments were performed three times in triplicate. Data are presented as mean ± SEM. The statistical analysis was performed by two-way ANOVA followed by Bonferroni's post hoc multiple comparison test (^∗^*P* < 0.05 versus non-Tg/placebo group; °*P* < 0.05 versus 3×Tg-AD/placebo group).

Finally, we wanted to explore if there was any impairment in neuronal functionality guided by astrocytes that is responsible for BDNF production [30]. To address this goal, we tested both BDNF and MAP2 expression. Western blot analysis showed an impaired production of BDNF in the FC of placebo-treated 3×Tg-AD animals compared to placebo-treated non-Tg littermates. Interestingly, um-PEA significantly counteracted such decrease (*P* < 0.05) (Figures 5(a) and 5(b)). Surprisingly, these modifications did not affect neuronal survival. Indeed, by both Western blot and immunofluorescence, we did not observe modifications in MAP2 expression in placebo-treated transgenic mice in comparison with placebo-treated non-Tg animals (Figures 5(a), 5(c), 5(d), and 5(e)).

Altogether, these results demonstrate the presence of reduced trophic support to FC neurons in 6-month-old 3×Tg-AD mice; this has not yet impaired neuronal viability. Chronic um-PEA treatment restores the neuronal trophic support.

## 4. Discussion

Astrocytes represent the crucial element of a defensive system of CNS. Cerebral insults trigger reactive astrogliosis, which represents a conserved defensive reprogramming of astroglial cells. It is a very heterogeneous phenomenon that limits damage and facilitates postlesion regeneration of neural networks [31–33], although it may become neurotoxic in some circumstances [34]. In fact, astrogliosis involves complex biochemical and functional remodelling and produces multiple reactive cellular phenotypes. The common feature of pathological astroglial change is the morphological cellular remodelling towards atrophy or hypertrophy [35, 36]. This sometimes occurs at early pathological stages preceding (and possibly precipitating) neuronal death. Such an early activation is observed in AD, when astrocytes, in proximity of plaques and *β*-amyloid deposits, acquire a reactive phenotype detectable by an increased expression of intermediate filaments and overproduction of proinflammatory mediators [23, 35, 37]. By this way, astrocytes contribute to neurodegeneration, becoming interesting targets for the development of innovative therapies [38–41]. Here, we provide the first *in vitro* evidence of the presence of a basal reactive state in primary astrocytes derived from 3×Tg-AD mice cortices, as well as of the capability of PEA to counteract such a phenomenon and improve neuronal viability. We also obtained important data on the beneficial pharmacological properties of this compound by testing the effect of a chronic treatment with um-PEA in 3×Tg-AD mice. Our *in vivo* results indicate that, during the mild stage of the disease, both reactive astrogliosis and neuroinflammation are already detectable in the FCs of transgenic animals, and that chronic um-PEA is able to alleviate both indices.

Several clinical studies have shown that an impairment of hippocampus, entorhinal cortex, posterior cingulate gyrus, amygdala, and parahippocampal gyrus occur in early AD [42–45]. When AD becomes severe, atrophy progresses from the hippocampus to the FC [46]. Interestingly, our results revealed the absence of neuronal atrophy in 6-month-old 3×Tg-AD mice (age that corresponds to a mild stage of pathology), but detected the early presence of reactive astrogliosis, confirming that such phenomenon is precocious and comes before neuronal loss in this animal model.

Since glial cells, previously considered only space-filling support cells of the CNS, are indeed highly involved in the maintenance of CNS homeostasis, we wondered whether cells in the FC were abnormally activated during the mild stage of the disease, before atrophy occurs. To this aim, we first performed an *in vitro* screening of 3×Tg-AD cortical astrocytes and compared them with non-Tg-derived cells, for signs of astrocyte activation and inflammation. In the absence of any exogenous insult, 3×Tg-AD primary astrocytes showed a basal reactive and proinflammatory phenotype, as demonstrated by the increased expression of GFAP and iNOS, a cytoskeletal astrocyte marker and a proinflammatory inducible enzyme, respectively. Since reactive gliosis may occur before plaque formation, these results suggest that astrocytes can contribute to AD progression before the development of the main AD hallmarks [40]. Since some astrocytes express little or no GFAP [47], we used an additional astrocytic marker, such as S100B, a Ca^2+^-binding protein. S100B is a neurotrophic factor that improves neuronal survival during CNS development [48]. In adulthood, levels of S100B increase after brain damage and can be neurotoxic and proinflammatory [49]. We did not find any genotypic difference in S100B expression, in our experimental condition. Therefore, it is possible that this protein maintains trophic functionality in the cell population analysed in the absence of an actual injury. Moreover, to further evaluate the inflammatory component in this animal model, we studied a proinflammatory enzyme, COX-2, responsible for prostanoid formation. Its involvement in the cascade of events leading to neurodegeneration in AD is still controversial [50]. COX-2 expression did not change in 3×Tg-AD cortical astrocytes, confirming the hypothesis that COX-2 induction could be connected with neurotoxicity [51], but this was not assessed in this *in vitro* study.

In the last few years, increasing evidence has confirmed the effectiveness of PEA treatment against inflammation in different models of neurodegeneration [15, 21, 52, 53]. Here, we tested the effectiveness of this compound on those parameters that we found to be influenced by the 3×Tg-AD genotype. Therefore, first we demonstrated the absence of astrocytic and neuronal toxicity of PEA at three different concentrations (0.01, 0.1, and 1 *μ*M). Then, we performed the neutral red assay, a viability test, in both primary astrocytes and primary neurons, confirming that PEA is not cytotoxic and fosters neuronal viability at the highest concentration investigated. This result agrees with the already proven neuroprotective effects of PEA in different preclinical models of neurological disorders [15, 54, 55]. In a surgical rat model of AD, we recently demonstrated the ability of PEA to counteract the reactive gliosis caused by A*β*_(1–42)_ hippocampal infusion [15]. Here, we demonstrated that PEA exerts this pharmacological effect in a transgenic model of AD, expanding the range of pathological targets treatable with this compound. In fact, the present *in vivo* experiments confirmed the therapeutic potential of chronic PEA administration. Moreover, such experiments were designed to simulate a clinic-like treatment schedule; for this reason, 3-month-old 3×Tg-AD and sex- and age-matched non-Tg animals were chronically treated for three months with placebo or um-PEA, a crystalline form on micrometric size, which improves its pharmacokinetics properties [24, 56]. Consistent with our *in vitro* observations, in the FC of 3×Tg-AD mice, we detected the presence of reactive gliosis in the mild stage of the disease, as shown by the increased transcription and expression of both GFAP and iNOS. The expression of both S100B and COX-2 were not affected by the genotype. Only S100B mRNA was increased in transgenic cortices. Interestingly, chronic um-PEA treatment dampened such alterations, confirming its effectiveness against reactive gliosis *in vivo*. Another interesting result was the detection of lower levels of BDNF in the FC of transgenic mice in comparison with non-Tg littermates, and, even more significant, the discovery of the ability of chronic um-PEA at increasing the expression of BDNF. Despite the fact that we found a decreased expression of BDNF together with the presence of reactive gliosis, known to be able to amplify CNS damages [14, 47], we did not detect any impairment of neuronal viability in FC of 3×Tg-AD mice. Such evidence was surprising because it is widely accepted that at 6 months of age these transgenic mice show early symptoms of AD-like pathology and behavioural alterations [23, 57–59]. However, since CNS impairment in AD travels broadly from hippocampus to FC, we can speculate that the massive alteration of this brain region has not occurred as yet, and neurons can somehow survive these insults. In fact, Castello and colleagues demonstrated that BDNF reduction does not exacerbate A*β* and tau pathology, but is a consequence of the pathology itself [60]. If this is true, the improvement in BDNF production that we found in mice after chronic um-PEA treatment can be a positive sign of the control that such a molecule has on the pathology in its entirety, and not only against reactive gliosis.

## 5. Conclusions

In the present study, we expand the knowledge on glial activity in a triple transgenic model of AD that closely mimics the main features of the pathology. Here, we provide the first evidence that 3×Tg-AD mice present signs of reactive gliosis in the FC at an early stage of the disease. Moreover, we demonstrate for the first time that acute PEA *in vitro*, as well as chronic um-PEA *in vivo*, may counteract such phenomenon, improving the trophic support to neurons, in absence of astrocytes and neuronal toxicity. By the virtue of its safety [61], and considering the growing body of evidence regarding its efficacy, we foresee a possible translation of the results collected in animal models into the clinical practice, in the near future.

## Figures and Tables

**Table 1:** Experimental conditions for immunofluorescence.

Primary antibody	Brand primary antibody	Primary antibody dilution	Secondary antibody	Brand secondary antibody
Rabbit *α*-GFAP	Abcam	1 : 1000 0.5% BSA in TBS/0.25% triton X-100	FITC conjugated goat anti-rabbit IgG (H+L) 1 : 200, 0.5% BSA in TBS/0.25% triton X-100	Jackson ImmunoResearch
Rabbit *α*-S100B	Novus Biologicals	1 : 250 0.5% BSA in TBS/0.25% triton X-100	TRITC conjugated goat anti-rabbit IgG (H+L) 1 : 200, 0.5% BSA in TBS/0.25% triton X-100	Jackson ImmunoResearch
Mouse *α*-MAP2	Novus Biologicals	1 : 250 0.5% BSA in TBS/0.25% triton X-100	TRITC conjugated goat anti-mouse IgG (H+L) 1 : 200, 0.5% BSA in TBS/0.25% triton X-100	Jackson ImmunoResearch

**Table 2:** Primer sequences used for RT-PCR.

Sequence of interest	Primer 5′→3′		Annealing temperature (°C)	Number of cycles
GFAP	Forward	GAAGAGGGACAACTTTGCAC	61	32
	Reverse	GCTCTAGGGACTCGTTCGTG		
S100B	Forward	TAATGTGAGTGGCTGCGGAA	63	32
	Reverse	CCTCACCAAGGGCTAAGCAG		
iNOS	Forward	CAAGCTGATGGTCAAGATCCAGAG	64	40
	Reverse	GTGCCCATGTACCAACCATTGAAG		
COX-2	Forward	GCTGTACAAGCAGTGGCAAA	62	30
	Reverse	CCCCAAAGATAGCATCTGGA		
GAPDH	Forward	GCTACACTGAGGACCAGGTTGTC	64	30
	Reverse	CCATGTAGGCCATGAGGTCCAC		

**Table 3:** Experimental conditions for Western blot from 3×Tg-AD mice astrocytes and FC.

Primary antibody	Brand primary antibody	Dilution	Secondary antibody	Brand secondary antibody
GFAP	Abcam	1 : 50000 5% milk in TBS-T 0.1%	HRP conjugated goat anti-rabbit IgG 1 : 30000 5% milk in TBS-T 0.1%	Jackson ImmunoResearch
S100B	Novus Biologicals	1 : 1000 5% BSA in TBS-T 0.1%	HRP conjugated goat anti-rabbit IgG 1 : 10000 5% BSA in TBS-T 0.1%	Jackson ImmunoResearch
COX-2	Cell Signaling	1 : 1000 5% milk in TBS-T 0.1%	HRP conjugated goat anti-rabbit IgG 1: 10000 5% milk in TBS-T 0.1%	Jackson ImmunoResearch
iNOS	Sigma-Aldrich	1 : 8000 1% BSA in TBS-T 0.1%	HRP conjugated goat anti-rabbit IgG 1 : 10000 1% BSA in TBS-T 0.1%	Jackson ImmunoResearch
BDNF	Santa Cruz	1 : 500 5% milk in TBS-T 0.1%	HRP conjugated goat anti-rabbit IgG 1 : 10000 5% milk in TBS-T 0.1%	Jackson ImmunoResearch
MAP2	Novus Biologicals	1 : 250 5% BSA in TBS-T 0.1%	HRP conjugated goat anti-rabbit IgG 1 : 10000 5% BSA in TBS-T 0.1%	Jackson ImmunoResearch
A*β*_(1–42)_	Millipore	1 : 1000 5% BSA in TBS-T 0.1%	HRP conjugated goat anti-mouse IgG 1 : 10000 5% BSA in TBS-T 0.1%	Jackson ImmunoResearch
*β*-actin	Santa Cruz	1 : 1500 5% milk in TBS-T 0.1%	HRP conjugated goat anti-rabbit IgG 1 : 10000 5% milk in TBS-T 0.1%	Jackson ImmunoResearch
